# To the Editors of the Medical and Physical Journal

**Published:** 1808-05

**Authors:** 


### *To the Editors of the Medical and Physical Journal.*

GENTLEMEN,

UPON reading the last Number of your Journal, I observed a letter signed *Harlestonensis,* in which some invidious sarcasms are thrown out against the use of mercury by the medical men in a neighbouring sea-port. Although your correspondent has taken the precaution to attack me under a mask, I can assure him, that it required very little penetration on my part to discover him: what motive he has for deprecating my practice, it is impossible for me to say, he has seen many cases *with me,* in which the use of mercury was *very successful,* and I am sure that I have always treated him in the most liberal and gentleman-like manner, both in and out of his profession; I believe I may say, without exaggeration, that I have been a sincere and useful friend to *Harlestonensis, and I am still willing to be his friend;* for when I consider his youth and irritable temper, I am rather inclined to pity than resent his present conduct. I only hope that he will, in future, remember that, Ira *furor* brevis est. As for my practice, I trust that I am able to defend it by correct and philosophical arguments; I hope that I am not bigoted to any particular medicine; but after having used *mercury* in many cases, for these twenty years past, with constant benefit to my patients and credit to myself, it would be strange indeed for me to dismiss it, to gratify the arrogant petulance of a juvenile practitioner.

I am, &c.

W. G. M. D.

*Yarmouth, April* 8, 1808.

